# Macular involvement in patients with Behçet’s uveitis

**DOI:** 10.1007/s12348-012-0075-9

**Published:** 2012-05-02

**Authors:** Rim Kahloun, Salim Ben Yahia, Samah Mbarek, Sonia Attia, Sonia Zaouali, Moncef Khairallah

**Affiliations:** 1 Department of Ophthalmology, Fattouma Bourguiba University Hospital, 5019 Monastir, Tunisia; 2 Faculty of Medicine and University of Monastir, Monastir, Tunisia

**Keywords:** Behçet’s uveitis, Macula, Macular edema, Optical coherence tomography, Visual impairment

## Abstract

**Purpose:**

The purpose of this study is to assess macular involvement in patients with Behçet’s uveitis.

**Methods:**

The study included 65 patients (120 eyes) with Behçet’s uveitis. All patients underwent detailed ophthalmic examination, including dilated biomicroscopic fundus examination, fundus photography, fluorescein angiography, and optical coherence tomography. Follow-up ranged from 6 to 46 months (mean 20 months).

**Results:**

At initial examination, 29 eyes (24.1 %) had macular involvement including macular edema (16 eyes, 13.3 %), serous retinal detachment (SRD; five eyes, 4.1 %), active retinitis (three eyes, 2.5 %), macular hole (three eyes, 2.5 %), macular atrophy (two eyes, 1.6 %), macular ischemia (one eye, 0.8 %), epiretinal membrane (one eye, 0.8 %), branch retinal vein occlusion involving the macula (three eyes, 2.5 %), and branch retinal artery occlusion involving the macula (two eyes, 1.6 %). During follow-up, 22 eyes (18.3 %) developed macular complications including macular edema (ten eyes, 8.3 %), SRD (four eyes, 3.3 %), active retinitis (two eyes, 1.6 %), severe macular atrophy (two eyes, 1.6 %), macular ischemia (three eyes, 2.5 %), macular hole (one eye, 0.8 %), epiretinal membrane (two eyes, 1.6 %), and subretinal fibrosis (one eye, 0.8 %). Branch retinal vein occlusion involving the macula developed in two eyes (1.6 %). Final best corrected visual acuity in patients with macular involvement ranged from 20/400 to 20/25 (mean 20/80).

**Conclusions:**

Macular edema and other vision-threatening macular complications are common in Behçet’s uveitis. Macular damage is often irreversible, causing permanent visual impairment. Early and appropriate treatment of Behçet’s uveitis is mandatory to reduce the risk of visual impairment due to macular involvement.

## Introduction

Behçet disease (BD) is a chronic multisystem disorder characterized by relapsing inflammation of unknown etiology. It is an important cause of morbidity throughout the world, with high prevalence in the Mediterranean, and Far and Middle Eastern countries. The frequency of ocular involvement in patients with BD disease is around 70 % [1]. The typical form of ocular involvement is a relapsing remitting uveitis involving more commonly both anterior and posterior segments.

Vitritis, retinal infiltrates, sheathing of predominantly retinal veins and occlusive vasculitis are the typical signs of posterior segment inflammation. Macular edema is a major cause of visual morbidity in patients with BD [1, 2]. Data on other maculopathies are scarce.

The purpose of our study was to assess macular involvement in patients with of Behçet’s uveitis.

## Materials and methods

The charts of 65 consecutive patients (120 eyes) with posterior or panuveitis secondary to BD examined at the department of Ophthalmology of Fattouma Bourguiba University Hospital, Monastir, Tunisia, were reviewed. The diagnosis of BD was made according to the International Study Group for Behçet's Disease criteria [3]. Posterior uveitis and panuveitis were defined according to the criteria of the Standardization of Uveitis Nomenclature Working Group [4].

All patients underwent detailed ophthalmic examination including measurement of Snellen best-corrected visual acuity (BCVA), slit-lamp examination, tonometry, and dilated fundus examination with noncontact and contact lenses, fundus photography, and optical coherence tomography (OCT) at initial examination and during follow-up. Fluorescein angiography (FA) were also performed for all patients at initial examination and when needed during follow-up. Mean follow-up was 20 months (range, 6–46 months).

## Results

Mean age at presentation was 34 years (range 16–57). Forty-eight patients (73.8 %) were male and 17 (26.2 %) were female.

At initial examination, 29 eyes (24.1 %) had macular involvement. In patients with macular involvement, initial best-corrected visual acuity ranged from light perception to 20/50 (mean 20/200).

**Fig. 1:**
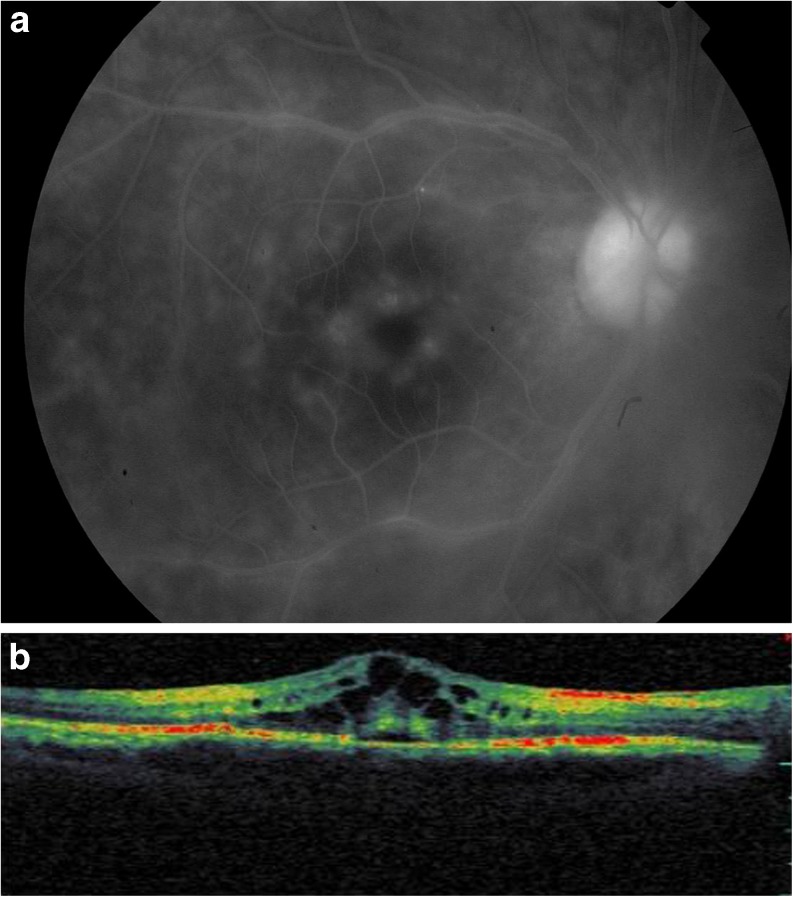
**a** Intermediate-phase fluorescein angiography of a 35-year-old female patient with Behçet disease shows cystoid macular edema with vascular leakage and optic disk hyperfuorescence. **b** Optical coherence tomography shows serous retinal detachment associated with the cystoid macular edema

Macular edema was noted in 16 eyes (13.3 %; Fig. 1). It was detectable on FA in 12 eyes (75 %), and only observed on OCT in four eyes (25 %).

Serous retinal detachment (SRD) was found in five eyes (4.1 %). It was detectable only by OCT in three eyes and visible on fundus examination in two eyes. SRD was related to branch retinal vein occlusion in three eyes and to corticosteroid-induced central serous chorioretinopathy in one eye. It was associated with cystoid macular edema in four eyes (3.3 %; Fig. 1).

**Fig. 2:**
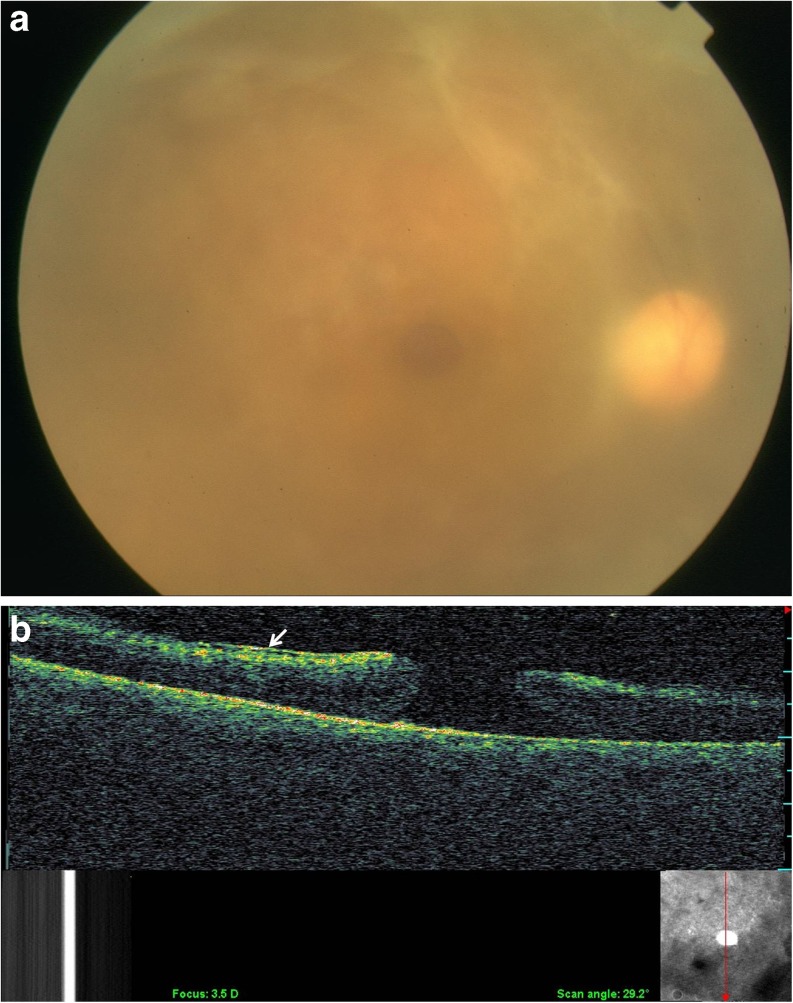
**a** Fundus photography of a 23-year old male patient with Behçet disease showing a macular hole. **b** Optical coherence tomography shows a full thickness macular hole with epiretinal membrane (*white arrow*)

Other findings included active retinitis in three eyes (2.5 %), macular hole in three eyes (2.5 %; Fig. 2), macular atrophy in two eyes (1.6 %), epiretinal membrane in one eye (0.8 %), and macular ischemia in one eye (0.8 %). Branch retinal vein and artery occlusion involving the macula were noted in three eyes (2.5 %) and two eyes (1.5 %), respectively (Table 1).

**Table 1:** Macular involvement in patients with Behçet’s uveitis

Types of macular involvement	At presentation		During follow-up	
	*N* = 29 of 120		*N* = 22 of 120	
			Eyes with initial macular involvement (*N* = 4)	
			Newly affected eyes (*N* = 18)	
	Number of eyes	%	Number of eyes	%
Macular edema	16	13.3	10	8.3
Serous retinal detachment	5	4.1	4	3.3
Active retinitis	3	2.5	2	1.6
Macular hole	3	2.5	1	0.8
Macular atrophy	2	1.6	2	1.6
Macular ischemia	1	0.8	3	2.5
Epiretinal membrane	1	0.8	2	1.6
Branch retinal vein occlusion	3	2.5	2	1.6
Branch retinal artery occlusion	2	1.6	–	–
Subretinal fibrosis	–	–	1	0.8

During follow-up, 22 eyes (18.3 %) developed macular complications. There were four eyes (3.3 %) with initial macular involvement and 18 (15 %) newly affected eyes.

**Fig. 3:**
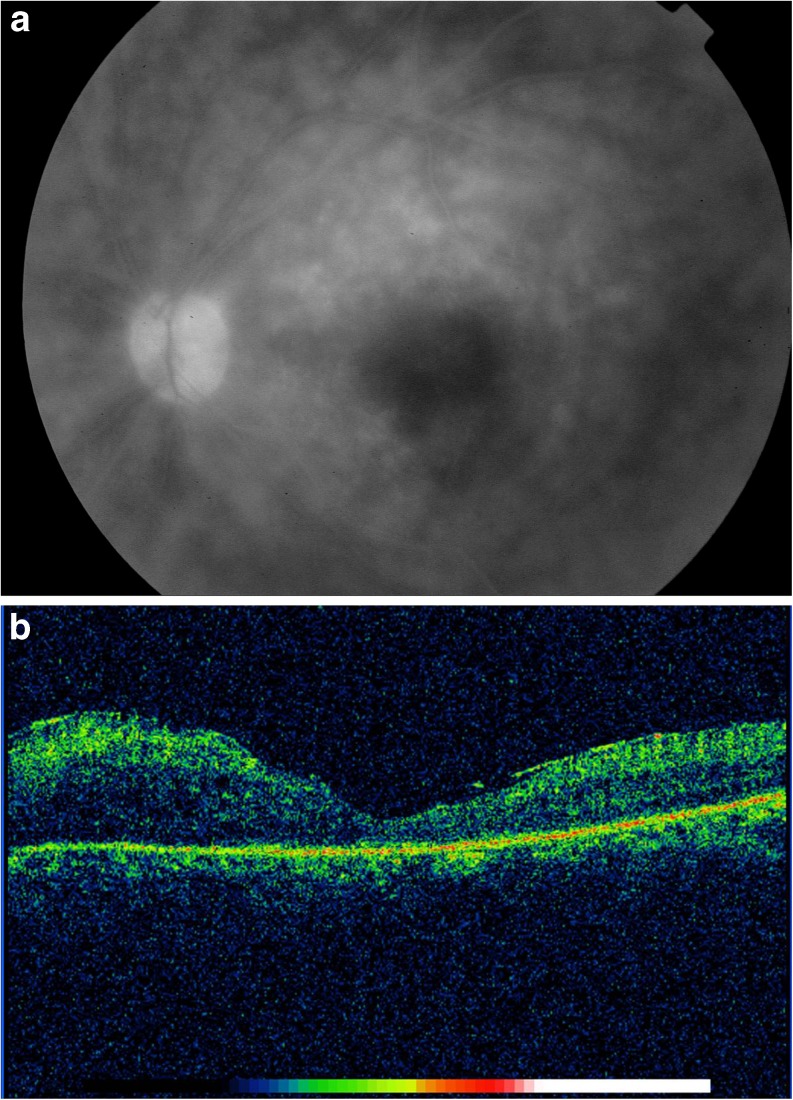
**a** Late-phase fluorescein angiography of a 33-year-old female patient with Behçet disease shows diffuse capillaritis. **b** Optical coherence tomography reveals macular atrophy with epiretinal membrane

Macular complications included macular edema (ten eyes, 8.3 %), SRD (four eyes, 3.3 %), active retinitis (two eyes, 1.6 %), macular ischemia (three eyes, 2.5 %), epiretinal membrane (two eyes, 1.6 %), and branch retinal vein occlusion involving the macula (two eyes, 1.6 %). Three eyes with initial macular edema developed severe macular atrophy (two eyes, 1.6 %; Fig. 3) and a macular hole (one eye, 0.8 %), respectively. One eye (0.8 %) with deep macular active retinitis developed subretinal fibrosis (Table 1).

All patients with macular involvement were treated with oral or intravenous corticosteroids associated with azathioprine (2.5 mg/kg/day). Ten patients (27.8 %) received in addition cyclosporine A (5 mg/kg/day).

Nine eyes (7.5 %) with macular edema were also treated with intravitreal injection of triamcinolone acetonide (4 mg in 0.1 ml), two eyes (1.6 %) with intravitreal injection of bevacizumab (1.25 mg in 0.05 ml), and one eye (0.8 %) with intravitreal injection of methotrexate (400 μg in 0.1 ml).

Two patients with macular hole underwent vitrectomy with internal limiting membrane peeling and gas tamponade. The macular hole was closed in one patient with improvement of visual acuity, and persisted for the second patient.

Final BCVA in patients with macular involvement ranged from 20/400 to 20/25 (mean 20/80). It was ≤20/200 in eight eyes (19 %) and was ≥20/40 in 20 eyes (47.6 %).

## Discussion

BD is a chronic disease characterized by recurrent attacks of severe inflammation that may cause significant ocular damage leading to irreversible alterations and significant vision loss.

Main causes of serious vision loss in Behçet’s uveitis include optic nerve atrophy, macular damage, and retinal vascular occlusions causing ischemic retinopathy with neovascularization, vitreous hemorrhage, or neovascular glaucoma [1, 2, 5, 6].

Macular involvement in Behçet’s uveitis may include macular edema, active focus of retinitis, SRD, severe macular atrophy, macular ischemia, macular hole, epiretinal membrane, vascular occlusion, and subretinal neovascularization [1, 2, 5–12].

In our series, macular involvement was detected in 24 % of eyes at presentation, and occurred in 18 % of eyes during follow-up. Macular edema is by far the most common complication, reported in 11.3 to 62 % of patients with Behçet’s uveitis [1, 2, 5, 7–10]. It can be the result of inflammatory process or the consequence of branch retinal vein occlusion, a common complication of Behçet retinal vasculitis. Macular edema can evolve into cystoid degenerescence, macular atrophy, and occasionally macular hole leading to permanent visual impairment.

SRD was the second common macular complication in our series. It was associated with cystoid macular edema and only detected by OCT in the majority of cases.

Macular hole is a rare complication of BD panuveitis and can ultimately lead to severe vision loss. This complication was reported in 2.6 and 3.4 % of patients with Behçet’s uveitis [1, 2]. It may be the consequence of macular edema or caused by changes on the vitreoretinal interface leading to vitreoretinal tractions due to recurrent intraocular inflammation [11].

Macular ischemia is associated with poor visual prognosis [4, 12]. It may be the consequence of occlusive retinal vasculitis involving veins or arteries.

Foci of active retinitis involving the macular area were also associated with visual loss due to macular atrophy and retinal pigment epithelium changes.

Poor visual acuity (≤20/200) was recorded in 19 % of patients with macular involvement in our series. It was associated with macular ischemia, severe macular atrophy, macular hole, and subretinal fibrosis.

In conclusion, macular edema and an array of other macular changes can occur in patients with Behçet’s uveitis. They can result in severe, often irreversible visual loss. Early and appropriate treatment of Behçet’s uveitis is mandatory to minimize the risk of visual impairment due to macular involvement.
